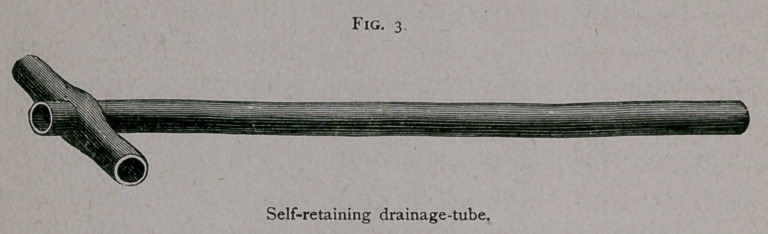# A Contribution to the Study of Pelvic Abscess

**Published:** 1889-02

**Authors:** Clinton Cushing

**Affiliations:** Professor of Gynecology, Cooper Medical College, San Francisco, Cal.; No. 636 Sutter Street


### *A CONTRIBUTION TO THE STUDY OF PELVIC ABSCESS.*[1]

By CLINTON CUSHING, M. D.,

Professor of Gynecology, Cooper Medical College, San Francisco, Cal.

It is with some hesitation that I venture to discuss a subject which is doubtless familiar to all the members of this association; nevertheless, it is one of great practical importance, and one that enters into the daily experience, more or less, of every busy man who has much to do with the treatment of diseases peculiar to women.

---

1. Read at the annual meeting of the American Association of Obstetricians and Gynecologists, in Washington, September 18, 1888.

The proportion of women who, at some time in their lives, have suffered from a pelvic abscess, is quite large. I am not aware that any statistics have been published by which we can judge accurately of the relative number of cases, but it is sufficiently great to warrant a chapter on this subject in most of the leading works on gynecology.

For the purposes of this paper, we will define a pelvic abscess to be any collection of pus in the cavity of the pelvis, which exists in the tissues outside the cavities of the hollow organs, such as the bladder, the uterus, the Fallopian tubes, and the rectum. When it is remembered that pelvic abscess is extremely rare in men, except as a consequence of direct violence, and that it is a very common affection in women, we are forced to the conclusion that the frequency of the disease in the female sex is due to the peculiar conformation and function of the pelvic organs, and this peculiarity is characterized by the functions of ovulation and menstruation; and, furthermore, by the fact that we have communication between the peritoneal cavity and the outside of the body by a direct canal.

During the past few years, I have published three series of cases of pelvic abscess that illustrate most of the causes which ordinarily produce this disease. Among them are cellulitis, caused by pyemic infection from amputation of the cervix, from a deposit of tubercle, by disease of the vermiform appendix, from a hematocele, pelvic peritonitis, pyosalpinx, and disease of the pelvic bones. Of these causes, none has proved so important and frequent as pyosalpinx.

The growth of our knowledge, in later years, regarding the diseases of the Fallopian tubes, and the somewhat extended experience in the observation and treatment of pelvic diseases, have led me to endorse, in the main, the views of Mr. Lawson Tait regarding the frequency of the existence of salpingitis and pyosalpinx; and, while it is doubtless true that the various causes already mentioned furnish us with a considerable proportion of examples of pelvic abscess, I have no doubt that a large proportion of recurrent pelvic inflammation, terminating in the formation of pus, is due to infection of the affected part by irritating secretion from disease of the Fallopian tubes.

It is only within the past few years that we have been made aware of the frequency of tubal disease by the numerous laparatomies, and the many post mortems undertaken with the object of ascertaining definitely the causes of the serious symptoms, or of the death of the patient. Bandl states that in the examination of one hundred uteri, he found catarrhal disease of the tubes in over half the cases. At the present day, all are aware of the frequency among all classes and conditions of women, of a chronic inflammation of the mucous membrane that lines the cervix and body of the uterus, and it is but fair to suppose that, as the uterine end of the Fallopian tube is continuous with the endometrium, by continuity the mucous membrane of the Fallopian tubes is also in a state of catarrhal inflammation, and that this condition constituting the predisposing cause, it only requires some untoward circumstance, such as the introduction of a uterine sound, the congestion which attends menstruation, unusual exercise, or the sexual act, to cause the expulsion of muco-purulent matter from the tube directly into the peritoneal cavity; and we here have the explanation of the unlooked-for attacks of pelvic inflammation which not infrequently follow a most careful examination of the cavity of the uterus, this examination occurring at the hands of a skilful and experienced gynecologist, in spite of every precaution being taken to avoid such an unfortunate accident.

I am more and more convinced each year of the statement made by Noeggerath, that an uncured gonorrhea in the male is a very frequent cause of salpingitis, a recent case coming under my observation, in which this theory was verified in every particular:

A woman, twenty-seven years old, who had been seduced five years before, since which time she had been a servant in the family of a physician, and had led, so far as could be ascertained, a moral life, had suffered during this entire time from chronic pelvic disease, resulting in repeated attacks of pelvic inflammation, incapacitating her at times from attending to her duties. Two months before I saw her she had married; immediately following the marriage all her symptoms became very much aggravated, and the husband contracted a severe urethritis, which resulted in a perineal abscess. The woman's condition became so serious that I was asked by her physician to see her. I diagnosticated pyosalpinx, with a probable pelvic abscess. I opened the abdomen, and found the right Fallopian tube distended with pus, and, at its outer extremity, a pelvic abscess containing several ounces. I removed both tubes and ovaries, and now, upon an examination by a competent microscopist, gonococci were found in both Fallopian tubes, intermingled with the pus. The secretion from the urethra of the husband was therefore examined, and the gonococci demonstrated without difficulty. Here, then, was a case where it would appear that the woman had been infected with the gonorrhea five years before; that the specific character of the disease had remained in the Fallopian tubes, where it had caused repeated attacks of pelvic inflammation; that the husband had contracted the disease of the woman; and that the sexual relations had aggravated the already existing disease of the woman, resulting in the formation of the pelvic abscess which necessitated the operation. The patient recovered without a bad symptom, and was able to attend to her duties in thirty days.

The pus cavity may consist of the distended Fallopian tube constituting pyosalpinx; it may be situated anywhere in the pelvic excavation, in the ovary, or in the peritoneal cavity at its lower part, bounded above by the small intestines agglutinated by lymph, a remarkable example of which came under my observation four years ago:

A girl, eighteen years of age, was suffering from some serious abdominal disease, which I diagnosticated as pelvic abscess. I opened the abdomen in the linea alba, and it was now found that that portion of the abdominal cavity below the umbilicus was an immense sac of pus, the upper wall of which was composed of the small intestines and mesentery, agglutinated together by lymph. The uterus was crowded backward and downward against the rectum; the ovary and Fallopian tubes were buried in a mass of lymph; the cavity contained at least a gallon of pus. A counter opening was made through Douglas's pouch, and a rubber drainage-tube drawn through from the abdominal opening into the vagina. On the second day, the bladder wall sloughed, and all the urine escaped into the pus cavity. This necessitated the formation of an artificial vesico-vaginal fistula, that the urine might drain away from below, in order to allow the pus cavity to heal. At the end of a year the abscess cavity had healed, the abdominal wound had closed, and having now repaired the vesico-vaginal fistula, the patient was restored to a perfect state of health.

Some cases have recently been reported by Martin, of Berlin, in which the collection of pus was situated in a deposit of lymph among the intestines which overlie the pelvic excavation. I have seen one case of this character. I have found that the most common point of exit of a pelvic abscess is into the rectum, next into the vagina, then through the abdominal wall, into the bladder, through the perineum, sometimes through the Fallopian tube into the uterus, into the peritoneal cavity in the order named, and occasionally, but very rarely, through the sacro-sciatic notch. An illustrative case, in which the exit of the pus was through the sacro-sciatic notch, occurred in the wife of a physician in this city, under my care, about three years ago. She was confined of her first child on July 1st; at the end of three days, puerperal fever developed, which was treated by her physician in the usual manner, with quinine and opium. The case went steadily on from bad to worse, and when I first saw her, on July 20th, her state was most deplorable; the temperature varied from 100° to 104°; the abdomen was moderately distended, and the right hip and thigh were enormously enlarged. There was not sufficient pain at this time to require opiates. In consultation with the physician in charge, I advocated the free use of the aspirator, to determine, if possible, whether there existed any collection of pus. This was opposed, on the ground that the woman's condition did not warrant such procedure. On the third day thereafter, however, an aspirating needle was introduced just above the trochanter, on the outer surface of the thigh, and, at a depth of an inch and a half, a pus sac was discovered. A free incision was now made, followed by the introduction of the finger, when it was found that the fascia lata was the outer covering of an immense pus cavity, and that the end of the finger could be passed down behind the trochanter major to the sides of the pelvis. It was clearly made out that the channel extending from the pus cavity into the pelvic excavation, or, in other words, that the pelvic abscess had bored through the sacro-sciatic notch into the tissues about the hip-joint. I now divided the tissues along the crest of the ilium, and then with two fingers pushed off the peritoneum from the iliac fossa, passed my fingers down into the pelvic cavity, and found that the periosteum had disappeared from the inner side of the ramus of the ischium—that there was, in fact, a condition of caries. A drainage-tube was introduced through the roof of the vagina, up through the abdominal opening, and the parts thoroughly washed out. The temperature immediately dropped to normal, and the prospects were very good for recovery; but the woman gradually sank, and died of exhaustion on the third day. Had this treatment been carried out earlier in the case, I doubt not that she would have recovered.

The diagnosis is sufficiently easy in many cases, but in others only the aspirator or abdominal section can make clear the facts. Certain symptoms have been set down as proof of the existence of pus, notably, persistent high temperature, chills, and night-sweats; and, as a rule, these symptoms, or some of them, are present, and are a reliable guide for opinion and practice. But I saw a physician's wife, two months ago, who was steadily failing in health and becoming much emaciated, having lost forty pounds in weight in the past five months, succeeding an attack of pelvic inflammation, which left masses of organized lymph about the uterus. There was great tenderness on pressure, but no signs of fluctuation, no increased temperature, and no rigors or night-sweats. I advised abdominal section, but was opposed by both physician and patient, and therefore, as a compromise, used the aspirating needle through the roof of the vagina; to the right of the uterus, at a depth of two inches, I found an abscess of small size, which was probably a Fallopian tube, containing thick, cheesy pus. The dilating trocar was used, and, through the opening made, a self-retaining drainage-tube was inserted: at this writing the discharge has nearly ceased, and the local and general symptoms much improved. I think there can be no doubt that a collection of pus may occur in the pelvis, may become encysted, the fluid portion gradually absorbed, and a cheesy mass be left behind, which may remain in *statu quo* for years, unless unfavorable general conditions, or local injuries, or irritations set in action new inflammatory processes, in which event we have the predisposing cause for the formation of an acute pelvic abscess. If the abscess be not due to the existence of tubercle or cancer, and if blood-poisoning is not present, under proper treatment, the prognosis is good.

At the present day, our methods have become so certain and direct that, given a case which still retains a fair share of vitality, the evacuation of the pus and the keeping of the parts afterward in a cleanly condition will insure a recovery.

The differential diagnosis, when made from a digital examination of the rectum or of the vagina, is often attended with great difficulty. A pelvic hematocele, or an extra-uterine pregnancy, may simulate a pelvic abscess. In the case of a hematocele, the sudden appearance of the tumor, the absence of firmly organized lymph, and the absence of high temperature, should be sufficient to arouse our suspicions of the existence of a blood-clot. In extra-uterine pregnancy, the previous history, the suspension of menstruation, the enlargement of the breasts, the absence of high temperature, nausea, and non-fixation of the uterus would be sufficient evidence on which to base an opinion; but in any case in which the health is seriously imperiled, or life in danger, we are justified, either with an aspirator through the roof of the vagina or by abdominal section, to determine positively the nature of the disease with which we have to deal.

In the treatment of a pelvic abscess, and I would include as well, because the two diseases are so often associated, pyosalpinx, the rule should be invariably that when it is manifest that we have to deal with an accumulation of pus which causes decided constitutional disturbance and local pain, the surgeon should use artificial means to evacuate the collection in the speediest and safest manner. When it is evident, from the repeatedly recurring attacks of pelvic inflammation coming on without any apparent cause, that pyosalpinx exists, abdominal section is, without doubt, the best method of treatment; it enables the surgeon to go clearly into the merits of the case, and to remove such diseased structures as may be needful to produce a radical cure. If, on the other hand, as a consequence of hematocele or a cellulitis, we have a collection of pus in which fluctuation can be made out from the vagina by digital examination, the puncture can be made through the vagina, and the pus cavity drained by a method which, in my hands, has proven very satisfactory.

About six years ago, I had under my care a case of pelvic abscess which gave me great anxiety. A collection of pus existed behind the uterus, which produced constitutional symptoms of so grave a nature that I feared for the life of my patient. Being satisfied of the existence of the abscess, I made a puncture directly behind the cervix into Douglas's pouch, and evacuated about three ounces of bad-smelling pus. The hemorrhage from this incision was so extremely profuse, that I was finally compelled to plug the opening with cotton which had been saturated with Monsel's solution; this, of course, for the time being, put a stop to any further escape of pus from the abscess; however, no further bleeding occurred, and the abscess was nearly well, when again severe symptoms developed; increased thickening took place in the left ligament, and again severe constitutional symptoms supervened. It now became evident that I had to deal with a collection of pus in the left broad ligament, directly in the line of the uterine artery and the ureter. Upon using the aspirator, the abscess was easily found, at a depth of about one inch from the vagina, but I did not dare to use a knife to enlarge the opening, on account of the near proximity to the uterine artery and the ureter. I now had constructed what I term a dilating trocar —an instrument that I herewith present for the inspection of the Association.[1]

As will be seen, it is similar to a uterine dilator, but with a trocar point. This instrument I forced along the track made by

1. Figs. 1 and 2, page 377.

the aspirating needle, and then, by closing the handles, the points were separated, and the tissues were torn in such a way as to leave an opening sufficiently large for the drainage-tube to be introduced easily, the operation being attended with a minimum amount of risk to the uterine artery and the ureter. Since that time, I have treated many cases of pelvic abscess in this manner, and, when they were not complicated with pyosalpinx, the results have been all that could be desired.

The self-retaining drainage-tube, as will be seen from the sample,[1] is of a T-shape, and is easily made in a moment from a piece of rubber tubing of a suitable size. The cross-section should

FIG. 1.

Dilating trocar closed.

FIG. 2.

Dilating trocar open.

be sewed in place with a needle armed with a strand of heavy waxed silk. It is introduced by pressing the ends of the cross-section down by the side of the tube and holding it there in the grasp of a pair of forceps; the tube is now carried into the position intended, and, by liberating the forceps, the ends of the cross-section spring out and make the tube self-retaining. To remove the tube, it is only necessary to make a moderately strong traction, when the short ends fold upward, and the tube is easily drawn out.

There are a few points in this connection worthy of consideration. In the first place, the tube should not be removed until

1. Fig. 3, page 378.

the discharge from the abscess has practically ceased, in order that we may avoid a too early closure of the opening, and hence, a reäccumulation of the pus. In the introduction of the aspirating needle, or the trocar, a careful digital examination will enable

FIG. 3.

Self-retaining drainage-tube.

us to avoid arteries of any considerable size near the vagina, as their pulsation can be made out. In introducing the aspirating needle, the trocar, or the drainage-tube, the speculum is unnecessary; on the contrary, the sense of touch will locate more surely the proper point for the introduction of the needle or trocar, than would the sense of sight.

After the evacuation of the pus, if the temperature does not become normal, an injection of hydronaphthol in water can be used once in five or six hours. A point of great interest now arises regarding the question whether we have to deal with a case of pyosalpinx pure and simple, or whether we have a collection of pus outside of the Fallopian tube, and a case of pyosalpinx as well. The method of procedure in the treatment of the case will depend very much upon our ability to make this differential diagnosis; for, if we have a collection of pus in Douglas's pouch, and in the connective tissues of the pelvis, consequent upon some local inflammation and independent of any disease of the tubes, the opening of the abscess from below through the roof of the vagina, and the introduction of the drainage-tube is without doubt the safest and best method of procedure. If pyosalpinx exists, a tapping of the abscess through the roof of the vagina or even the tubes themselves, with an aspirator, and the subsequent introduction of a drainage-tube through an opening made with a knife, or with the dilating trocar, will not result in a cure.

The history of the case, rather than the information gained from a digital examination, will be our safest guide in cases of doubt. If it can be shown that there has been a series of attacks of pelvic abscess, coming on without apparent cause, and especially if a pelvic abscess has existed before, the chances are all in favor of the case being one of pyosalpinx, in which event the proper treatment is to open the abdomen and remove the diseased tubes; and I think that it may be set down as a rule that if a pelvic abscess, that is not tubercular, is opened in the manner already described and completely drained, and it then fails to heal, the probabilities are that we are dealing with pyosalpinx; and, further, that opening the abdomen will in the end become a necessity to effect a radical cure.

In several instances, I have found the best plan of treatment to be, after opening the abdomen, to make a puncture through the roof of the vagina with a dilating trocar, and insert a self-retaining drainage-tube; and, with the hand in the abdominal cavity, to control and govern the direction and depth of the opening made through the roof of the vagina. When the small intestines are agglutinated with masses of lymph, in the center of which exists a collection of pus, the advantage of the dilating trocar over the knife is self-evident; the risk of hemorrhage is much decreased; the torn tissues do not so readily reunite, and drainage is thus more certainly secured.

I would commend to the notice of the members of the Association the dilating trocar, for it has proven in my hands a most satisfactory instrument, enabling me in many cases to give speedy relief where, without it, procrastination would have been necessary and much valuable time lost. I would urge also the free use of the aspirating needle in all cases of obscure pelvic disease, in which the local or constitutional symptoms would indicate the presence of pus; I use it constantly in such cases, and never in an instance has its employment been followed by bad results.

No. 636 Sutter Street.

## Figures and Tables

**Fig. 1. f1:**
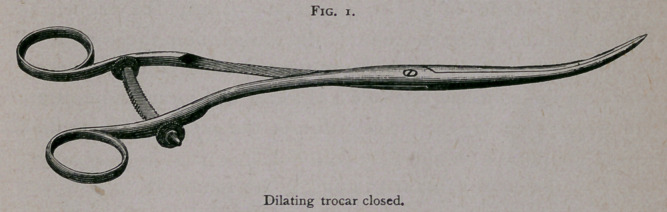


**Fig. 2. f2:**
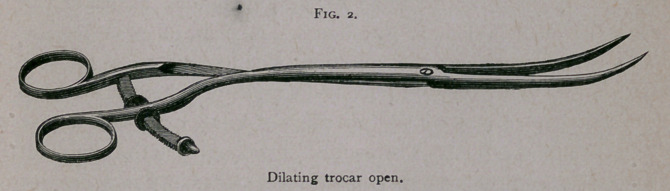


**Fig. 3. f3:**